# Integrated transcriptome analysis of jejunum and liver to identify key genes and pathways associated with body weight in chickens

**DOI:** 10.1038/s41598-025-34620-0

**Published:** 2026-01-27

**Authors:** El-Sayed M. Abdel-Kafy, Asmaa M. Elfiky, Neama I. Ali, Mohamed H. Abdelfatah, Esraa M. Abdel-Mageed, Fatma M. Behiry, Yasmein Z. Abdel-Ghafar, Yasmein Z. Abdel-Ghafar, Shereen. S. Ghoneim, Nada S. El-Shahwy, Sabbah F. Youssef, Hoda A. Shabaan, Huazhen Liu, Wael A. H. Ali

**Affiliations:** 1 Animal Production Research Institute (APRI), Agricultural Research Center (ARC), Dokki, P.O.12651, Giza, Egypt; 2 Department of Environmental and Occupational Medicine, Environment and Climate Change Research Institute, National Research Centre, Dokki, P.O.12622, Giza, Egypt; 3 Department of Cell Biology, National Research Centre, Biotechnology Research Institute, Dokki, P.O.12622, Giza, Egypt; 4 Department of Basic Veterinary Medicine, College of Animal Science and Veterinary Medicine, Huazhong Agricultural University, Wuhan, 430070 China

**Keywords:** Chicken, Transcriptome, Differentially expressed genes (DEGs), Pathways jejunum, Liver, Genetics, Molecular biology

## Abstract

This study investigates the transcriptomic variations in the jejunum and liver of Golden Montazah (GM) chickens to better understand the biological mechanisms influencing poultry growth and production. Given the vital role of poultry in fulfilling global protein demands, especially with the rising consumer preference for chicken, it is essential to explore these underlying genetic and molecular factors that drive growth. From a larger cohort of 480 GM chickens, the top 10 males in both the high-weight (HW) and low-weight (LW) groups were selected for RNA sequencing. Tissues from their jejunum and liver were collected for transcriptomic analysis. The results revealed 38 up- and 36 down-regulated genes in the jejunum, while the liver exhibited 109 up- and 74 down-regulated genes. Among these, notable differentially expressed genes (DEGs) such as *CHST14* and *LOC429682* in the jejunum, alongside *RBP2* and *STC2* in the liver, appeared to be integral to growth regulation, immune response, and metabolic processes. Functional enrichment analyses using GO and KEGG pathways highlighted processes like cytokine-cytokine receptor interactions in the jejunum and steroid biosynthesis in the liver. Additionally, protein–protein interaction networks identified key hub genes essential for various biological functions. Overall, our findings emphasize the distinct gene expressions profiles associated with body weight in the jejunum and liver, providing valuable insights for genetic improvement in poultry breeding. Understanding these molecular mechanisms paves the way for targeted strategies to enhance growth performance in the poultry industry.

**Supplementary Information:**

The online version contains supplementary material available at 10.1038/s41598-025-34620-0.

## Introduction

Animal production is vital for providing protein nutrition to the rapidly growing global population, with the poultry industry being a significant sector focused on cost-effective meat production. Chicken is favored for its tender meat quality and nutritional profile, leading to increased demand. Key performance indicators in poultry production, such as body weight, are vital measures of animal health and growth, directly impacting economic viability^1^.As the poultry industry faces new challenges, understanding physiological dynamics is essential for optimizing production practices and sustainability in food supply. Enhancing poultry production in the context of global warming increasingly relies on the utilization and development of local chicken breeds^2^. Many of these breeds may not have undergone intensive selection processes like some foreign breeds, which could contribute to their unique genetic traits and potential resilience^3,4^.

Investigating molecular mechanisms related to growth performance is crucial, as many key genes influence growth traits, though their regulatory processes remain unclear^5^. RNA sequencing (RNA-seq) provides a cost-effective and efficient method for detecting genetic variants that are likely to influence phenotypic traits, facilitating advancements in breeding strategies for improved poultry production^6^. Recent studies have leveraged RNA-seq to explore differentially expressed genes (DEGs) in chicken that linked to traits such as meat quality^7–11^, disease resistance^12,13^, and growth performance^6,11,14^. Digestive efficiency, a highly heritable trait, plays a crucial role in determining feed conversion^15^. The jejunum, a vital section of the small intestine, ensures effective nutrient absorption and serves as the first line of defense against pathogens^16^, ensuring the health and growth of the birds^17^. Furthermore, the liver plays a complex role in metabolism and immune response^18^ impacting digestion, cholesterol regulation, and vitamin production. It synthesizes proteins and regulates hormones crucial for production while contributing to metabolic homeostasis^18–20^. Recent studies utilizing RNA sequencing (RNA-seq) have provided valuable insights into gut biological processes in chickens, highlighting key aspects related to gene expression, metabolic pathways, and immune responses^10,18,21,22^. Also, RNA-seq significantly advanced our understanding of liver’ biological processes, revealing valuable insights into developmental stages, maternal effects, environmental adaptations, and metabolic pathways in chickens^8,18,23^.

Golden Montazah (GM) chickens hold significant importance in Egypt, particularly for smallholder poultry production, due to their remarkable disease resistance, ability to adapt to various climates, and impressive growth performance^24^. As one of the three officially recognized native Egyptian strains—alongside Silver Montazah and Mandarah—GM chickens are widely used in rural and small-scale poultry systems^25^. While large-scale commercial enterprises are dominated by breeds like Cobb and Ross, local breeds such as GM chickens are vital for ensuring food security in rural communities. Their resilience, capacity to thrive under low-input conditions, and role in conserving genetic diversity make them invaluable for sustainable poultry farming^25,26^. Despite their importance, the molecular mechanisms driving their exceptional growth remain largely elusive. Most existing transcriptomic research has focused on other breeds, leaving a notable gap in our understanding of GM chickens’ biology^27^. Also, to the best of our knowledge, no data has been published on the differential transcriptome between jejunal and liver tissues in chickens with high versus low body weights.

To address this gap, our study investigates potential candidate genes associated with high body weight and explores how these genes interact within the core network linking the jejunum and liver in GM chickens. Using integrated transcriptomic analysis, we aim to shed light on the genetic factors underpinning their growth, which could support more sustainable and tailored breeding strategies of local chicken production in Egypt.

## Methods

### Ethical approval and study location

This study was approved by the Animal Care and Use Committee at the Animal Production Research Institute (APRI), under the ethical approval number 2023393429, and in accordance with the "Principles of laboratory animal care" (NIH publication No. 86–23, revised 1985) and the ARRIVE guidelines. All methods were performed in accordance with the relevant guidelines and regulations. The field study was conducted at the APRI poultry research farm in El-Azab, Fayoum governorate, Egypt, while the laboratory analyses were performed at National Research Centre, Dokki, Giza.

### Bird management, housing and environmental conditions

A total of 480 Golden Montazah chickens were housed in battery cages, consisting of 20 cages with 24 birds each, in a brooder pen maintained at a constant temperature of 30 °C for the first three days. Following this initial period, the temperature gradually decreased by 3 °C each week until it reached 24 °C. Temperature control was automated using a thermostat, while ventilation was managed manually. Throughout the study, the chickens had ad libitum access to a mash diet and fresh water. From days 1 to 20, the birds were fed a starter diet containing 3025 kcal/kg of metabolizable energy and 21.5% crude protein. After day 21, they transitioned to a grower diet providing 3175 kcal/kg of metabolizable energy and 18% crude protein for the remainder of the study. These nutrient compositions were calculated based on NRC guidelines (1994). The lighting schedule began with 24 h of light for the first three days, followed by 20 h of light until day 7, before being reduced to 16 h thereafter. On their first day at the hatchery, all birds were vaccinated against Newcastle disease, infectious bronchitis, and Marek’s disease.

### Experimental design and sample collection

On the day of hatching, all birds were wing-banded and weighed, with equal numbers of males and females. At 49 days of age, the 20 heaviest (High Weight, HW) and the 20 lightest (Low Weight, LW) chickens were selected from the entire group of 480 GM chickens, representing 10 males and 10 females in each weight category to ensure balanced representation, based on their body weight rankings. Body weight gain was calculated as the difference between the initial body weight at hatching and the final body weight at 49 days. From these groups, only 10 males from each were selected for tissue sampling. After slaughter, the chickens were eviscerated, removing the gastrointestinal tract and internal organs—and their carcasses were weighed. The breast and leg muscles, including thigh and drumstick, were carefully dissected and weighed separately. Samples of jejunal mucosa and liver tissue were then collected and stored at -80 °C for further analysis.

### RNA extraction, library construction, and RNA-Seq

Samples of jejunal mucosa and liver tissue were collected from the 10 heaviest and 10 lightest males for RNA extraction. Total RNA was extracted from each using Trans-Zol Reagent (Transgen, Cat. #ET101) following the manufacturer’s instructions. To evaluate RNA integrity, the Agilent Bioanalyzer 2100 system (Agilent Technologies, USA) was utilized. Only RNA samples with a RIN of 7 or higher were selected for constructing cDNA sequencing libraries from the jejunum and liver samples using the NEBNext® Ultra™ RNA Library Prep Kit. The resulting cDNA libraries were sequenced by Bioss Biotechnologies in China using Illumina HiSeq platform.

### Differentially expressed gene (DEG) and function annotation analyses

The quality of raw read data for each sample was assessed using FastQC^28^. Low-quality reads (Q value ≤ 20), along with adaptors sequences, were trimmed using Trimmomatic (Bolger et al., 2014). The trimmed reads were then mapped to the *Gallus gallus* reference genome (GRCg6a, GCA_000002315.5) using HISAT2^29^. Mapped reads were quantified based on exons using the *Gallus gallus* GTF annotation file (galGal6.ensGene.gtf) with feature Counts^30^. Differential expression analysis for the Jejunum and liver, each consisting of 20 birds, was conducted using EdgeR v3.34.1^31^, following data normalization with the trimmed mean of M-values (TMM). Differentially expressed genes (DEGs) were identified with a Log2 fold change > 2 and a P-value < 0.05. Top set of most significant gene was selected by Benjamini–Hochberg method on the p-value. All expressed gene values and P-values were visualized in a volcano plot using the ggplot2 R package^32^. Multidimensional scaling (MDS) was performed to illustrate the similarity among samples for each tissue type. Additionally, heatmaps of the normalized read counts of DEGs were generated using the pheatmap R package^33^. Common and unique DEGs between tissue samples were represented using a Venn diagram created with Venny (https://bioinfogp.cnb.csic.es/tools/ venny/). To determine the biological functions associated with the DEGs, the Kyoto Encyclopedia of Genes and Genomes (KEGG) pathways and Gene Ontology (GO) terms (Cellular Component: CC, Molecular Function: MF, Biological Process: BP) were analyzed using the g:Profiler (https://biit.cs.ut.ee/gprofiler/gost)^34^ and ShinyGO (http://bioinformatics.sdstate.edu/go) databases^35^. A false discovery rate (FDR) < 0.05 was considered indicative of significant enrichment.

### Gene set enrichment analysis (GSEA)

The GSEA was conducted on all expressed genes, regardless of their differential expression status. The analysis utilized GSEA software from the Broad Institute (http://software.broadin stitute.org/gsea/index.jsp) and the C2.CP: KEGG gene set collections from the Molecular Signatures Database (MSigDB v7.0, Broad Institute, Cambridge, MA, USA)^36^. To assess the significance of gene expression differences between HW and LW groups in the jejunum and liver tissues, GSEA ranked all expressed genes. The enrichment score for each gene set was calculated using the complete ranked list of expressed genes, and the normalized enrichment score (NES) was determined for each gene set. Gene sets were considered significantly enriched based on the following criteria: absolute NES values > 1, nominal P-values ≤ 0.05, and false discovery rates (FDR) ≤ 0.05^37^.

### Protein–protein interactions network

The protein–protein interactions (PPIs) network, encompassing both direct and indirect relationships between proteins, was analyzed using STRING (http://string-db.org/)^38^. After evaluating the STRING results and the expression changes for each differentially expressed gene (DEG), a network diagram was constructed for the selected DEGs (those connected with one or more other DEGs) using Cytoscape v3.10.0^39^.

### Multi-omics factor analysis (MOFA)

The MOFA2 v1.8.0 package was employed to conduct Multi-Omics Factor Analysis (MOFA), an unsupervised statistical method designed to integrate various types of omics data^40^. Additionally, we used the Reactome Pathway Knowledgebase (https://reactome.org) to identify genes involved in protein metabolism^41^.

### Validation of RNA-seq through quantitative real-time PCR (qRT-PCR)

Total RNA was extracted from each sample using the Trans-Zol Reagent (Transgen, Cat. #ET101), following the manufacturer’s instructions. RNA integrity was assessed using 1% agarose gel, and RNA concentration and purity were determined with NanoDrop1000 (Thermo Scientific, Wilmington, DE, USA). First-strand complementary DNA (cDNA) was synthesized using RQ1 RNase-Free DNase (Promega, Cat. # M6101) and RevertAid First Strand cDNA Synthesis Kit (Thermo Fisher Scientific, Cat.# K1622) according to the manufacturer’s guidelines. After synthesizing cDNA from 1 μg of total RNA, qRT-PCR was conducted to validate the expression levels of five randomly selected DEGs identified in the RNA-seq analysis for both the jejunum and liver tissues.

Primers were designed using Primer-BLAST (https://www.ncbi.nlm.nih.gov/tools/primer -blast/) and synthesized by Synbio technologies (Table 1). The qRT-PCR was conducted using the QuantStudio 5 Dx Real-Time PCR System (Thermo Fisher Scientific) with Maxima SYBR Green qPCR Master Mix (2X) (Thermo Fisher Scientific, Cat. # K0251). Each qPCR reaction was performed in a final volume of 15 μL, containing 0.75 μL cDNA, 0.6 μL of each forward and reverse primer (10 μM), 7.5 μL 2 × SYBR green Master Mix, and 5.5 μL RNase-free ddH2O. Samples were run in triplicate. The quantitative PCR program consisted of an initial denaturation at 95.0 °C for 10 min, followed by 40 cycles of 95.0 °C for 15 s, 60.0 °C for 30 s and 72.0 °C for 30 s, concluding with a melting curve program consisting of 1 cycle at 95.0 °C for 15 s, 60.0 °C for 1 min, 95.0 °C 0.1 s. All samples were run in triplicate, with negative controls (no template) included on the same plate. The mRNA levels of DEGs were normalized to the housekeeping genes β-Actin. Relative gene expression values were calculated using the 2 − ΔΔCt method^42^. Finally, correlations between RNA-Seq data of the selected DEGs and the mRNA expression levels obtained from qRT-PCR were assessed to validate the RNA-seq results.

**Table 1:** The primer’s sequences used in the present study.

Gene	Forward primer	Reverse primer	Product length	Accession number
*Beta-Actin*	GACTGACCGCGTTACTCCCA	AGATGGGAACACAGCACGGG	128	NM_205518.2
*CALB1-201*	TACGACTCCGACGGCAATGG	ATTCTCCTCCGTCGGCAACA	195	NM_205513.2
*CHST14-201*	ATGACGCCGGACGAGATCAA	ACTCGGAGAAGGTCACGTCG	205	NM_001407339.1
*FADS6-201*	CTGCCATTACTGCCTGCTGC	GATCAGCCGCAAACATGGGG	152	XM_426241.8
*HSPB9-202*	CGTCTTCTGCTGAGAGGAGTG	CCGTTGTTCCGTCCCATCA	114	NM_001010842.3
*SGK2-203*	GAGCGTTGTTTCCGTGAGCC	AACACCACATGTCCCTGGCA	143	XM_046930972.1
*ACCS*	TCCGACGAGGAGGGGTACAA	AGCTGTTCCTTTAGCCGTTTGG	137	XM_015287196.4
*IRS2*	GTCCAGGAGAAAACCTATGCTTGG	ACGCTGTCCTCTCTCTTGTTCT	105	XM_015277882.4
*RBP2*	AAGGACTCGATAACCGGGTGG	ATCCAGTGCTTCCAGCCACG	106	NM_001277417.1
*SPTBN5*	GGTGAGCGGCTGAAAGATGC	TGTTCCAGGGCCACATGGTT	161	XM_040673837.2
*STC2*	GGGGCACATGGGATCATGGA	GCAGCCTTTGTCACTGCGTT	143	XM_040683047.2

#### Statistical analysis

All productive data were represented as means ± SEM with using Generalized Linear Model (GLM) technique in SAS software (2002 version, SAS Institute Inc., Cary, NC, USA). A t-test was employed to compare means, with differences deemed significant at *P* < 0.05. Statistical analyses were conducted on the 10 heaviest and 10 lightest males from both the high- and low-weight groups of GM chickens.

## Results

### Body weight, daily body weight gain and carcass weight

**Fig. 1:**
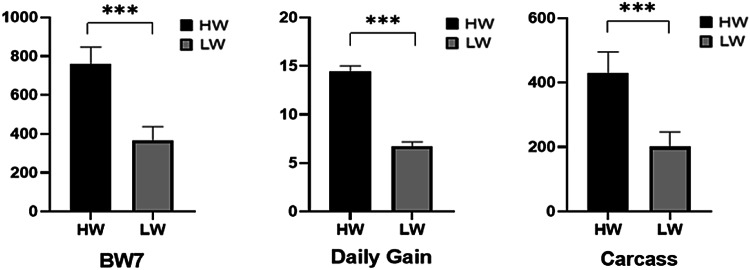
Final body weight at 7 weeks of age (BW7), daily body weight gain (Daily gain), and carcass weight (carcass) between high weight (HW) and low weight (LW) birds, significant at *P* < 0.05.

The final body weight at 7 weeks of age (BW7) and daily body weight gains for the top 10 males in both the high-weight (HW) and low-weight (LW) groups showed significant differences (*P* < 0.05), as illustrated in Fig. 1. Additionally, carcass weight also varied significantly between the groups (Fig. 1).

### Sequencing data and differentially expressed genes in the jejunum and liver

**Fig. 2:**
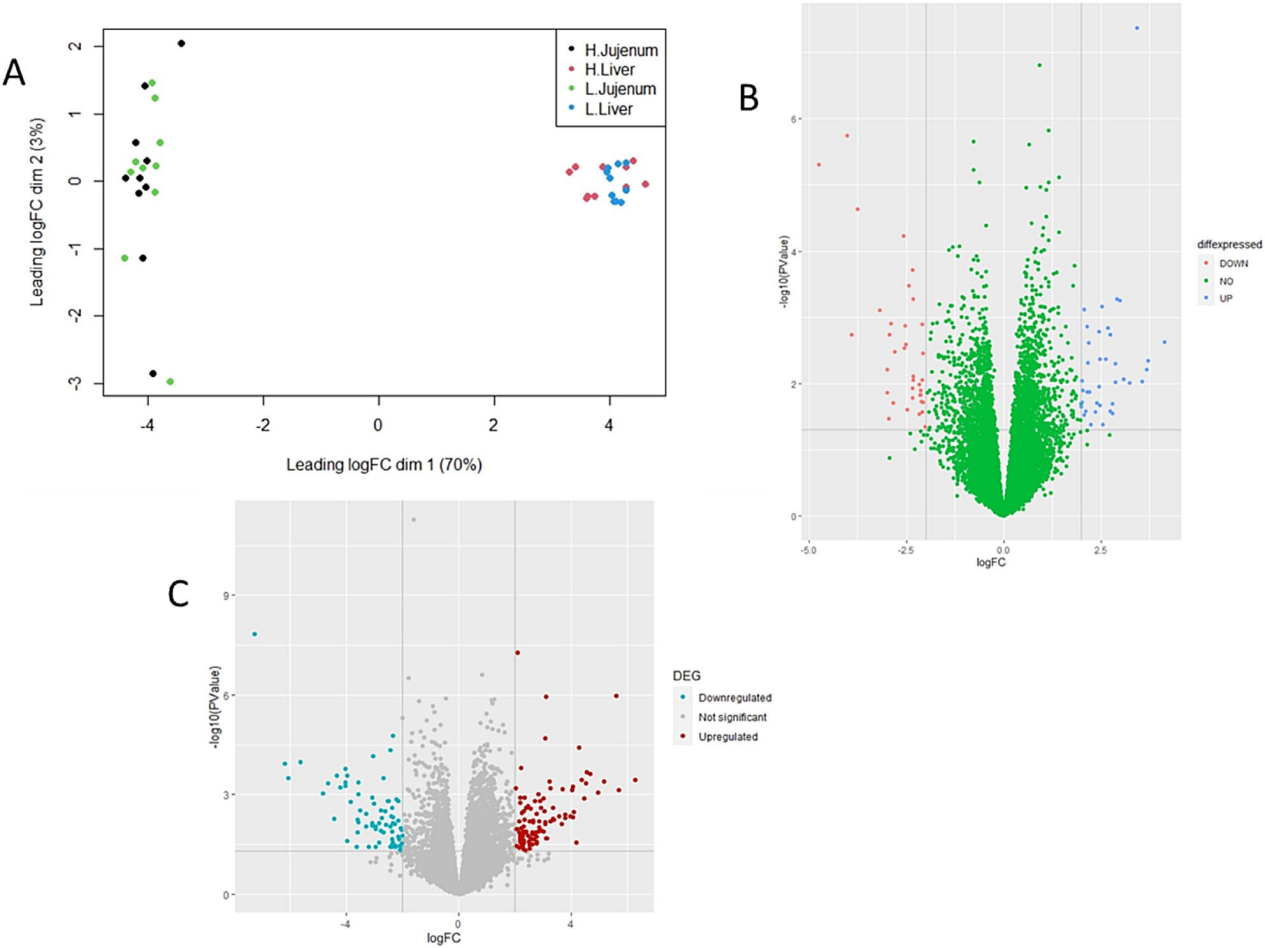
Overall transcriptomes in jejunal and liver tissues. (**A**): Multidimensional scaling (MDS) shows distinct clusters between the two tissues based on the transcriptomes of different weight groups (HW & LW). (**B**): Volcano plots reveal significantly differently expressed genes (DEGs) in the jejunum. (**C**): Volcano plots indicate significantly differently expressed genes (DEGs) in the liver. The x-axis represents log2 fold changes, while the y-axis represents log10 *P*-values.

Transcriptome sequencing of libraries from the jejunum and liver produced a total of 21.13 million and 20.78 million reads, respectively (Supplementary files, Table S1). Differential expression analysis between the HW and LW groups for each tissue was conducted using Edge-R, following data normalization. Multidimensional scaling (MDS) analysis revealed distinct clustering for each tissue (Fig. 2A).

Using a fold change threshold greater than 2 and a *P*-value of less than 0.05, we identified 38 genes that were upregulated and 36 that were downregulated in jejunal tissue when comparing the high-weight (HW) and low-weight (LW) groups (Supplementary Files, Table S2, and Fig. 2B). In liver tissue, the number of differentially expressed genes was higher, with 109 genes upregulated and 74 downregulated between the same groups (Supplementary Files, Table S3, and Fig. 2C). Tables 2 and 3 present the top 20 differentially expressed genes (DEGs) in the jejunum and liver in these groups, respectively, ranked by their log fold change (Log FC).

**Table 2:** Top 20 differentially expressed genes in the Jejunum between high and low weight groups, ranked by their log2 fold change (Log2 FC).

Gene		Log FC	*p*-Value	FDR	Trend
*CHST14*	Carbohydrate sulfotransferase 14	3.41	4.21E-08	0.0006	Up
*LOC429682*	GTPase IMAP family member 7-like	1.40	7.57E-06	0.0116	Up
*SGK2*	Serine/threonine kinase 2	1.40	5.11E-05	0.0358	Up
*FADS6*	Fatty acid desaturase 6	1.15	9.04E-06	0.0116	Up
*CD3E*	CD3e molecule	1.14	1.50E-06	0.0058	Up
*HCLS1*	Hematopoietic cell-specific Lyn substrate 1	1.09	1.19E-05	0.0119	Up
*LIMD1*	LIM domains containing 1	1.08	3.04E-05	0.0266	Up
*LSP1P1*	Lymphocyte-specific protein 1 pseudogene 1	1.02	4.44E-05	0.0327	Up
*PLEKHA2*	Pleckstrin homology domain containing A2	0.99	5.76E-05	0.0371	Up
*TACC1*	Transforming acidic coiled-coil containing protein 1	0.93	1.08E-05	0.0119	Up
*UNC119B*	Unc-119 lipid binding chaperone B	0.92	1.55E-07	0.0011	Up
*SEPTIN6*	Septin 6	0.65	2.47E-06	0.0058	Up
*GNAI3*	G protein subunit alpha i3	0.57	1.10E-05	0.0119	Up
*USP12-like*	Ubiquitin specific peptidase 12-like	−0.61	9.09E-06	0.0116	Down
*HNF1A*	HNF1 homeobox A	−0.77	5.87E-06	0.0103	Down
*HSPD1*	Heat Shock Protein Family D (Hsp60) Member 1	−0.78	2.22E-06	0.0058	Down
*CALB1*	Calbindin 1	−2.57	5.83E-05	0.0371	Down
*SULT6B1L*	Sulfotransferase family, cytosolic, 6B, member 1-like	−3.74	2.35E-05	0.0219	Down
*HSPB9*	Heat Shock Protein Family B (Small) Member 9	−4.01	1.80E-06	0.0058	Down
*LOC772158*	Heat shock protein 30C-like	−4.74	4.89E-06	0.0098	Down

**Table 3:** Top 20 differentially expressed genes in the liver between high and low weight groups, ranked by their log2 fold change (Log2 FC).

Gene		Log FC	*p*-Value	FDR	Trend
*RBP2*	Retinol Binding Protein 2	5.58	1.05E-06	0.0019	Up
*STC2*	Stanniocalcin 2	3.11	1.12E-06	0.0019	Up
*IRS2*	insulin receptor substrate 2	2.10	5.33E-08	0.0002	Up
*DUSP16*	Dual specificity phosphatase 16	1.56	7.87E-06	0.0054	Up
*MTSS1*	MTSS1, I-BAR domain containing	1.26	1.34E-06	0.0019	Up
*GALE*	UDP-galactose-4′-epimerase (GALE)	1.19	1.77E-06	0.0021	Up
*GPCPD1*	Glycerophosphocholine phosphodiesterase 1	1.15	6.36E-06	0.0049	Up
*NFKBIA*	NFKB inhibitor alpha	0.99	3.59E-06	0.0034	Up
*SIMC1*	SUMO interacting motifs containing 1	0.82	2.45E-07	0.0009	Up
*PRPSAP2*	Phosphoribosyl pyrophosphate synthetase associated protein 2	0.77	7.08E-06	0.0052	Up
*EMC8*	ER membrane protein complex subunit 8	−0.48	1.30E-06	0.0019	Down
*NFE2L2*	Nuclear factor, erythroid 2 like 2	−0.64	8.13E-06	0.0054	Down
*SRP14*	Signal recognition particle 14	−0.87	3.26E-06	0.0033	Down
*SCAP*	Cleavage activating protein (SCAP)	−0.94	2.22E-06	0.0024	Down
*PLPP7*	Inactive Phospholipid Phosphatase 7	−1.15	5.97E-06	0.0049	Down
*FDPS*	Farnesyl diphosphate synthase	−1.42	1.49E-06	0.0019	Down
*ARRDC2*	arrestin domain containing 2	−1.61	5.21E-12	0.00001	Down
*NECAB2*	N-terminal EF-hand calcium binding protein 2	−1.77	3.04E-07	0.0009	Down
*ACCS*	1-aminocyclopropane-1-carboxylate synthase homolog	−2.00	5.02E-06	0.0044	Down
*SPTBN5*	Spectrin Beta, Non-Erythrocytic 5	−7.26	1.49E-08	0.0001	Down

In jejunal tissue, the high-weight group exhibited 38 upregulated and 36 downregulated genes. According to false discovery rate (FDR) P-value, all the top 20 DEGs were significantly different (Table 2). Notably, *CHST14*, *LOC429682* and *SGK2* were identified as the most upregulated genes in the high-weight birds compared to their low-weight counterparts. Conversely, *LOC772158* and *HSPB9* showed significant downregulation (Table 2).

In liver tissue, a total of 109 upregulated and 74 downregulated genes were identified in the high-weight group. The top 20 DEGs are listed in Table 3. Among these genes, *RBP2* and *STC2* exhibited the highest levels of upregulation, while *ACCS* and *SPTBN5* displayed the greatest downregulation in high-weight birds, as indicated by their lower fold changes (Log FC) (Table 3).

**Fig. 3:**
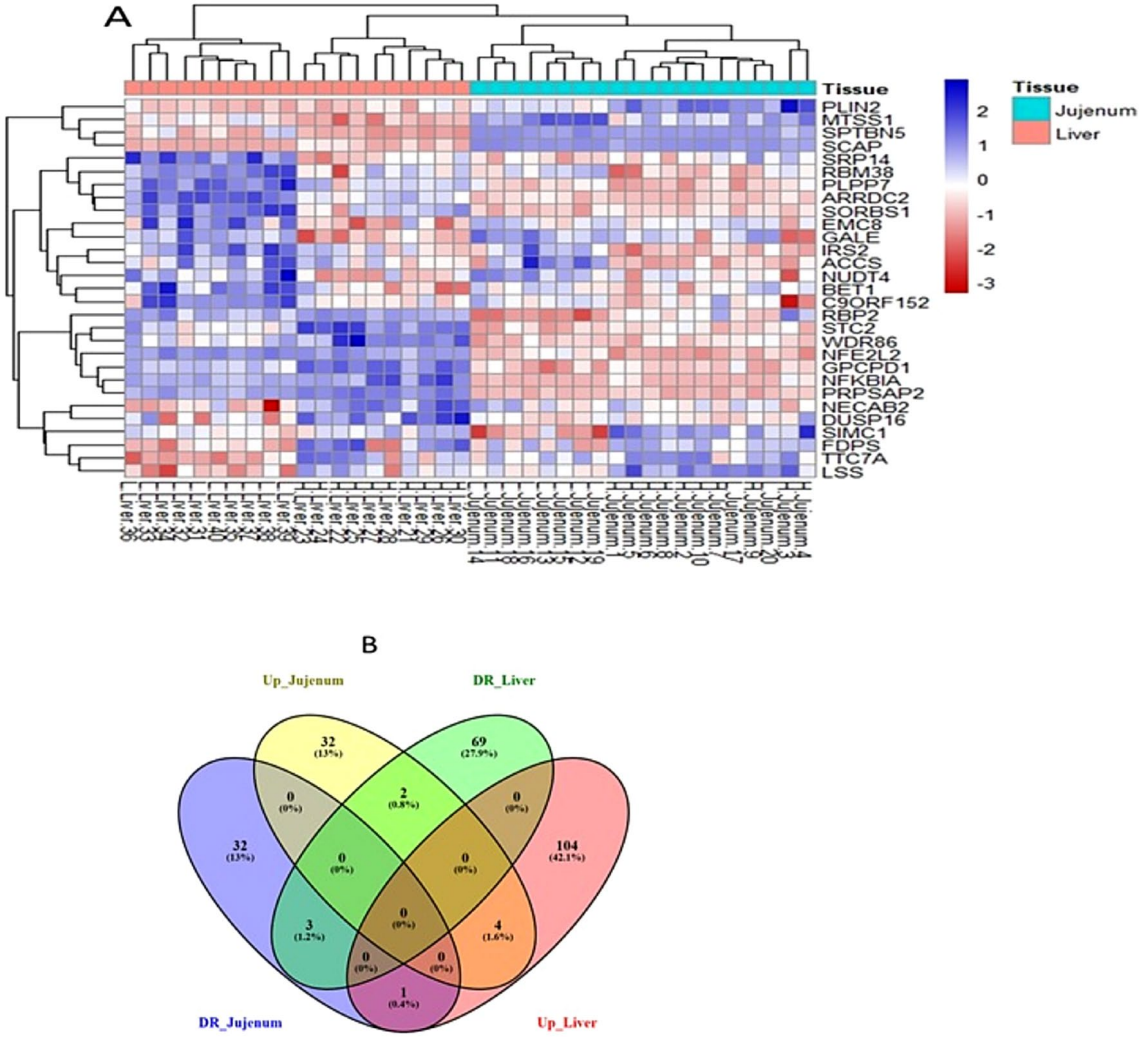
A: Heatmap illustrating differentially expressed genes (DGEs) at FDR < 0.05 among ten replicates of jejunum and liver tissues; expression values are transformed to log2. B: Venn diagram showing DGEs between jejunum and liver tissues, with “DR” indicating downregulated genes and “Up” indicating upregulated genes the high weight (HW) and low weight (LW) groups.

To visualize the relative abundance of significant genes across different tissues in high-weight birds compared to the low weight ones, a heatmap of differentially expressed genes (DEGs) was generated for all 40 samples (Fig. 3A). A Venn diagram illustrated the overlapping DEGs between the tissues (Fig. 3B), which included four up-regulated genes (ENSGALG00000046177, ENSGALG00000049716, ENSGALG00000048362, and ENSGALG00000053074), three downregulated genes (heat shock protein family B (small) member 9, ENSGALG00000050544, and ENSGALG00000042963), and three genes exhibiting reverse regulation. Specifically, two genes were upregulated in the jejunum (*ENSGALG00000041258* and peptide methionine sulfoxide reductase (*MsrA*)) but downregulated in the liver (glycoprotein nmb (GPNMB). In contrast, one gene was downregulated in the jejunum (*ENSGALG00000032282*) and upregulated in the liver (calbindin 1 (*CALB1*)) **(**Fig. 3B). Functionally, *CALB1* encodes a vitamin D-dependent calcium-binding protein crucial for calcium homeostasis, supporting skeletal development and muscle function. Its expression may influence body weight and growth-related processes.

### Gene ontology and KEGG pathway analysis in each tissue

**Fig. 4:**
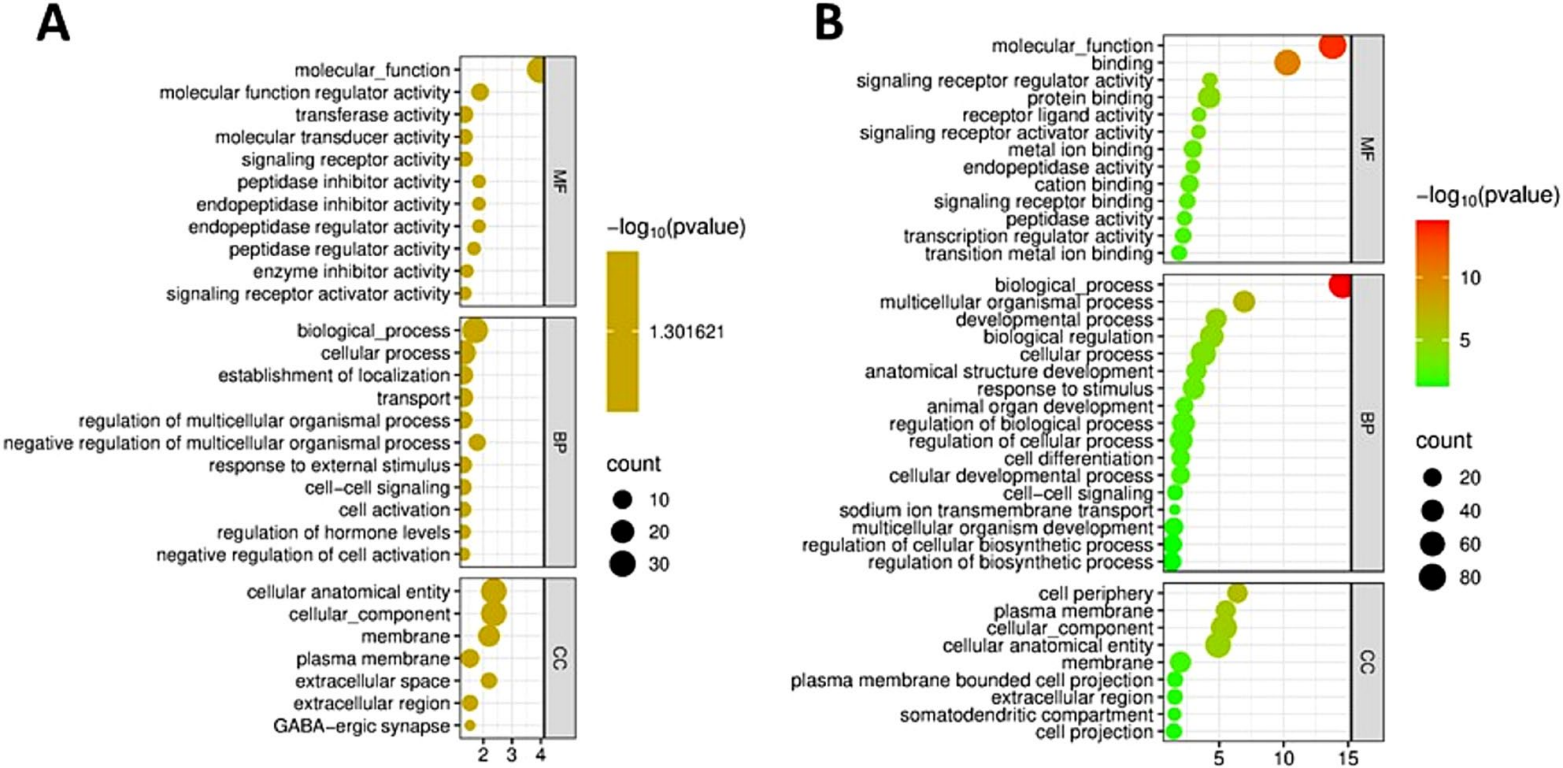
Gene ontology analysis of differentially expressed genes (DEGs) in HW and LW groups. (**A**): Gene ontology terms for DEGs in the jejunum. (**B**): Gene ontology terms for DEGs in the liver.

To explore the biological processes occurring in the studied tissues, a gene ontology analysis of differentially expressed genes (DEGs) was performed. The functional annotations and related genes were categorized into three groups: molecular function, cellular component, and biological process. In the jejunum, the most significant gene ontology categories (adjusted *P*-value < 0.05) included molecular function (GO: 0,003,674), molecular function regulator activity (GO: 0,098,772), biological process (GO: 0,008,150), cellular process (GO: 0,009,987), cellular anatomical entity (GO: 0,110,165) and cellular component (GO: 0,005,575) (Fig. 4A, Supplementary files, Table S4). In the liver, significant gene ontology categories similarly included molecular function (GO: 0,003,674), binding (GO: 0,005,488), biological process (GO: 0,008,150), multicellular organismal process (GO: 0,032,501), cell periphery (GO: 0,071,944), and plasma membrane (GO: 0,005,886) (Fig. 4B, Supplementary files, Table S4).

KEGG pathway analysis revealed significant enrichment of the cytokine-cytokine receptor interaction pathway (*p* < 0.05) in jejunal tissue (Table 4), when comparing high and low weight groups. This pathway involved key genes such as Activin receptor type IIB (*ACVR2B*), Growth Differentiation Factor 2 (*GDF2*), and Tumor Necrosis Factor Receptor Superfamily Member 13C (*TNFRSF13C*).

**Table 4:** Enriched KEGG pathway of differentially expressed genes (DEGs) in each tissue for each group.

Tissue	KEGG pathway	Adjusted *p-* value	Count	Genes
Jejunum	Cytokine-cytokine receptor interaction	0.0348	3	Activin receptor type IIB (*ACVR2B*), Growth Differentiation Factor 2 (*GDF2*), Tumor Necrosis Factor Receptor Superfamily Member 13C (*TNFRSF13C*)
Liver	Neuro-active ligand-receptor interaction	0.0443	6	Neuropeptide Y (NPY), Cholinergic Receptor Muscarinic 5 (*CHRM5*), Islet amyloid polypeptide (*IAPP*), G protein-coupled receptor 83 like (*GPR83L*), G protein-coupled receptor 25 (*GPR25*), Motilin receptor gene (*MLNR*)
	Steroid biosynthesis	0.0496	2	Cytochrome P450 family 24 subfamily A member 1 (*CYP24A1*) and Lipase member M-like 5 (*LIPML5*)

In liver tissue, the analysis identified significant (*p* < 0.05) enrichment in the neuroactive ligand-receptor interaction pathway and the steroid biosynthesis pathway (Table 4) in high weight birds compared to the low weight. The neuroactive ligand-receptor interaction pathway included six DEGs: Neuropeptide Y (*NPY*), Cholinergic Receptor Muscarinic 5 (*CHRM5*), Islet Amyloid Polypeptide (*IAPP*), G Protein-Coupled Receptor 83 (*GPR83L*), G Protein-Coupled Receptor 25 (*GPR25*), and Motilin Receptor (*MLNR*). Meanwhile, the steroid biosynthesis pathway analysis highlighted the involvement of genes such as Cytochrome P450 family 24 subfamily A member 1 (*CYP24A1*) and Lipase member M-like 5 (*LIPML5*) (Table 4).

### Gene set enrichment analysis

To compare gene expression levels between the high weight (HW) and low weight (LW) groups in each tissue, we conducted Gene Set Enrichment Analysis (GSEA). We identified significantly enriched gene sets, with a false discovery rate (FDR) of less than 0.05 for each tissue (Supplementary files, Table S5). Based on the KEGG-based list, positive and negative normalized enrichment scores (NES) indicate higher and lower expression levels, respectively.

**Fig. 5:**
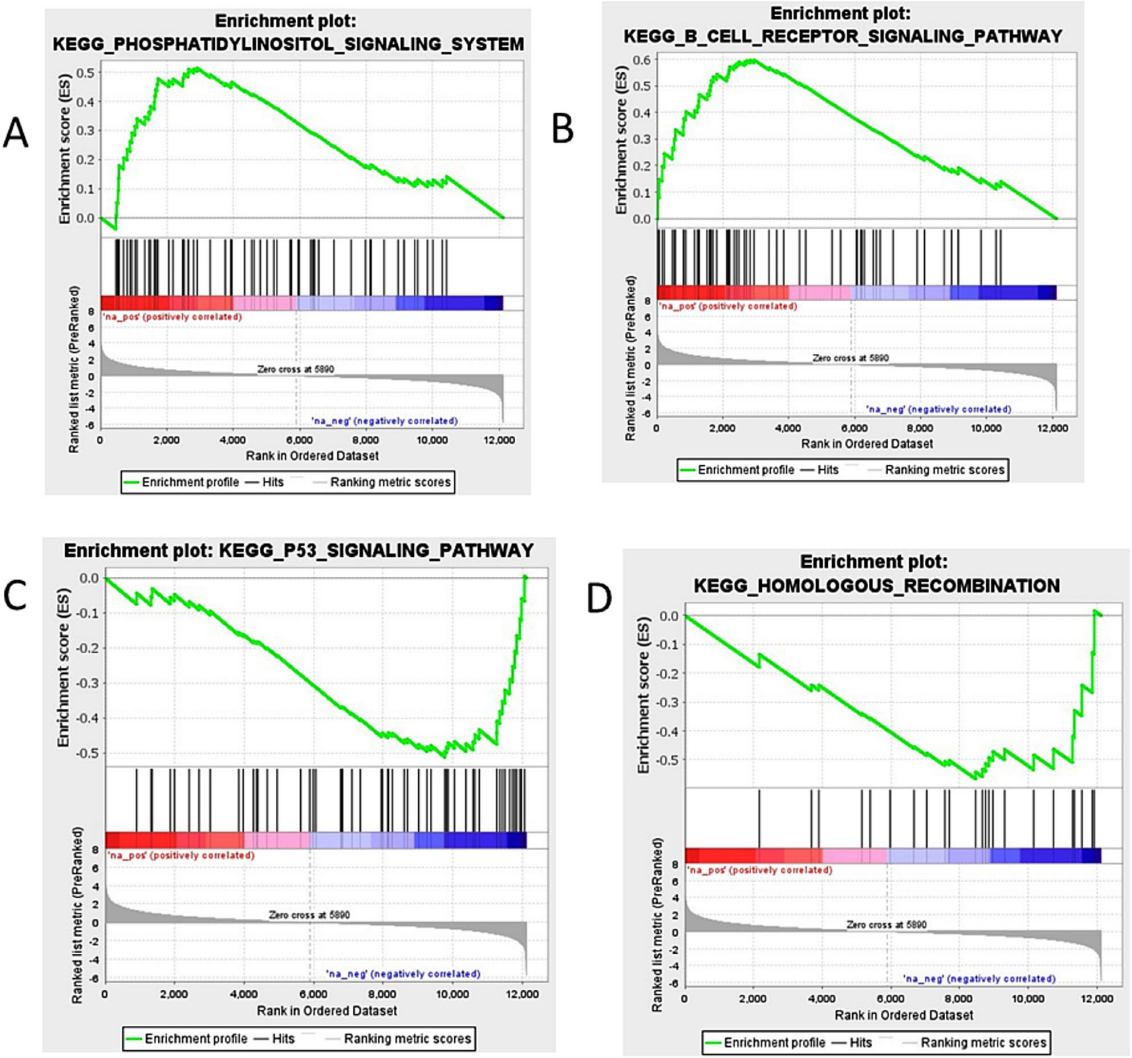
Gene set enrichment analysis (GSEA) performed between the HW and LW groups in jejunal tissue. (**A**) & (**B**): Gene sets with positive enrichment scores (ES). (**C**) & (**D**): Gene sets with negative enrichment scores (ES).

In jejunal tissue, the gene sets with higher expression included the ‘Phosphatidylinositol Signaling_System’ and ‘B_Cell _ Receptor_Signaling_ Pathway’ (Fig. 5 A, B). Conversely, the gene sets with lower expression encompassed the ‘p53_Signaling_Pathway’ and ‘Homologous_Recombination’ (Fig. 5 C, D).

**Fig. 6:**
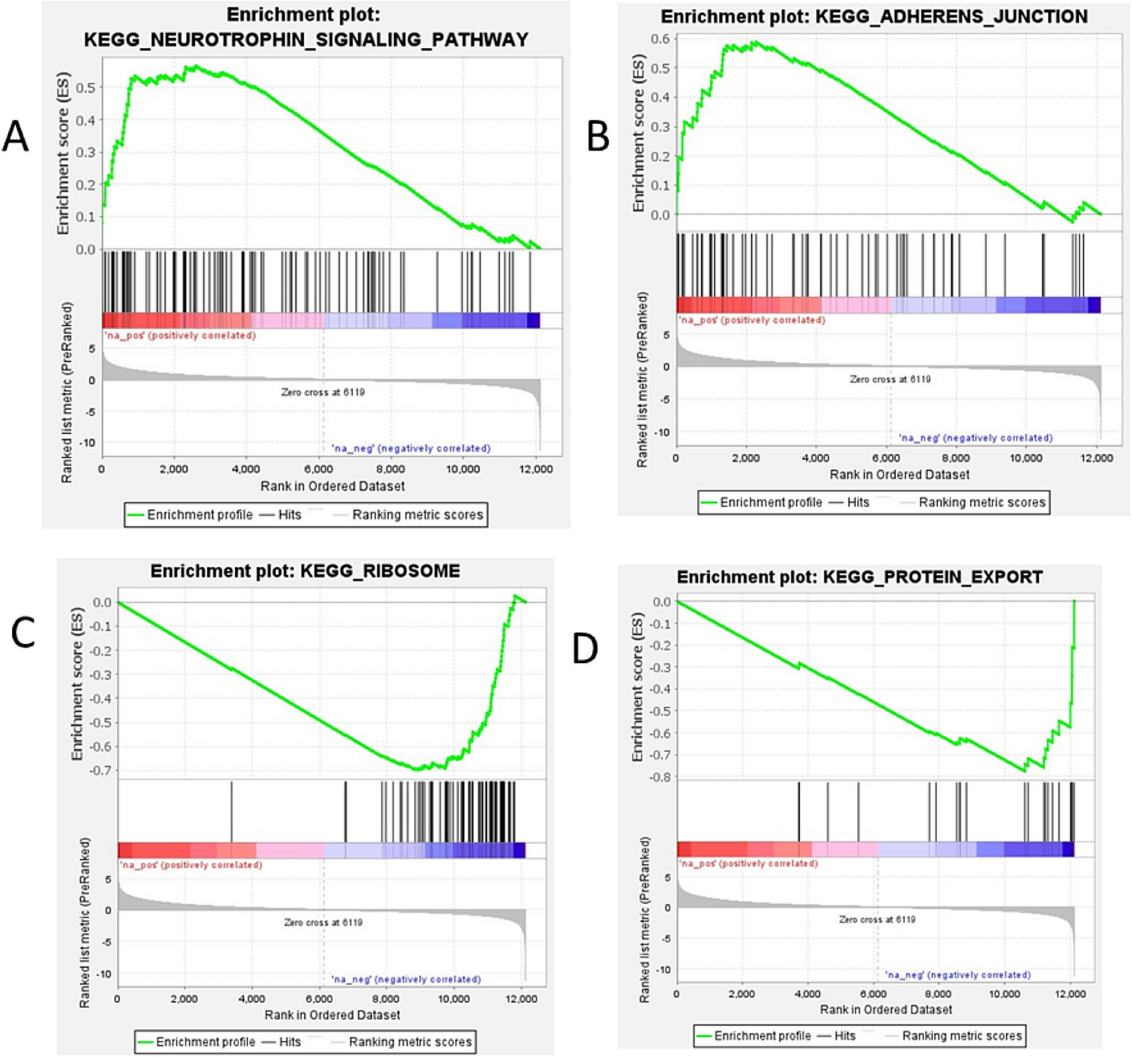
Gene set enrichment analysis (GSEA) performed between the HW and LW groups in liver tissue. (**A**) & (**B**): Gene sets with positive enrichment scores (ES). (**C**) & (**D**): Gene sets with negative enrichment scores (ES).

In liver tissue, the gene sets exhibiting higher expression included the ‘Neurotrophin_ Signaling_Pathway’ and ‘Adherens_Junction’ in the birds were the high weight birds (Fig. 6 A, B). In contrast, the gene sets with lower expression were ‘Ribosome’ and ‘Protein_Export’ (Fig. 6 C, D).

### Protein–protein interactions (PPIs) analysis of DEGs

**Fig. 7:**
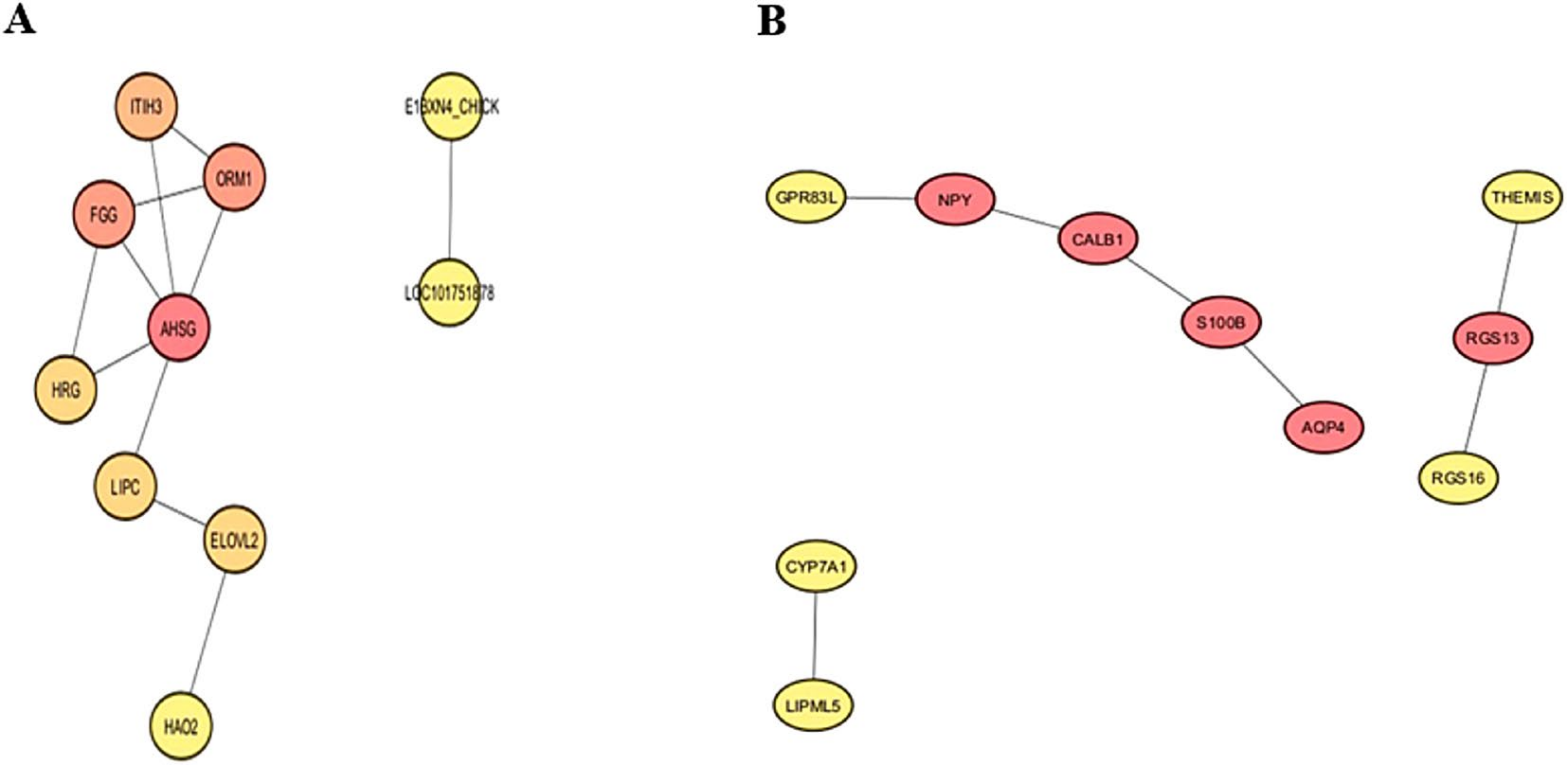
Identification and ranking of hub genes based on Maximal Clique Centrality (MCC) in (**A**) Jejunum and (**B**) Liver in HW and LW groups. Node color intensity reflects MCC scores: red indicates highly ranked hub genes (high MCC score), yellow represents low-ranking genes (low MCC score), and intermediate shades denote medium-ranking nodes.

Comparing high- and low-weight birds, our analysis identified the top 10 hub nodes in jejunal tissue, which were as follows: alpha 2-HS glycoprotein (AHSG), fibrinogen gamma chain (FGG), orosomucoid 1 (ORM1), inter-trypsin inhibitor heavy chain 3 (ITIH3), hierarchical random graph (HRG), lipase (LIPC), elongases of very long chain fatty acids 2 (ELOVL2), avian β-defensin 9 (AvBD9), hydroxyacid oxidase 2 (HAO2), and neuromedin U receptor 2 (E1BX N4) (Fig. 7A).

Similarly, in liver tissue, the top 10 hub nodes included neuropeptide Y (NPY), regulator of G protein signaling 13 (RGS13), aquaporin 4 (AQP4), S100B protein, calbindin 1 (CALB1), islet amyloid polypeptide (IAPP), proprotein convertase subtilisin/kexin type 1 (PCSK1), cytochrome P450 family 7 subfamily A member 1 (CYP7A1), lipase member M-like 5 (LIPML5), and thymocyte selection-associated (THEMIS) (Fig. 7B).

### Multi-omics integration through factor analysis

**Fig. 8:**
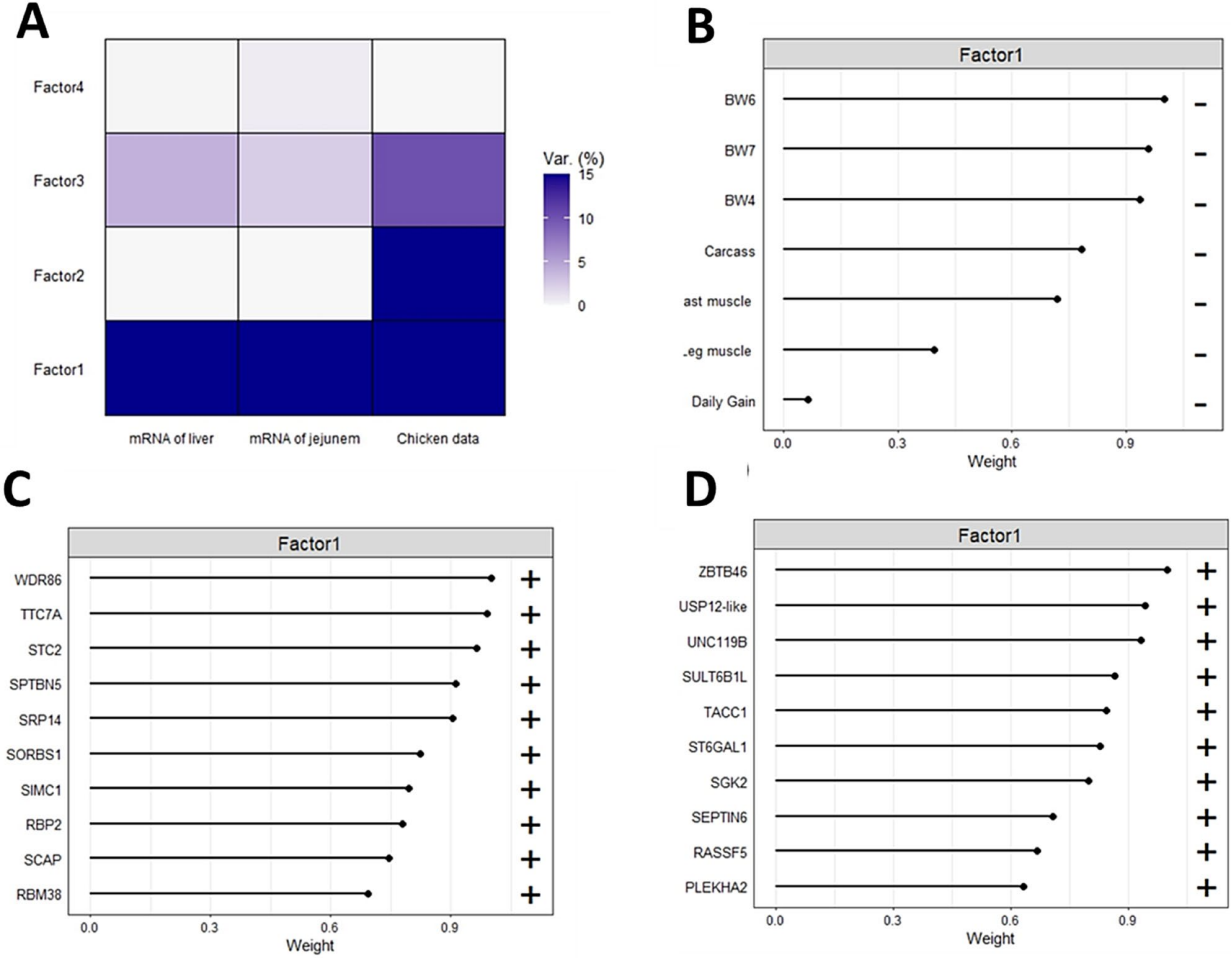
Integrative analyses of mRNA data from jejunum and liver. (**A**): Proportion of total variance explained (R2) by individual factors for each assay, showing absolute loadings of the top features of Factor 1 across all data. (**B**): Absolute loadings of the top features of Factor 1 in chicken data. (**C**): Absolute loadings of the top features of Factor 1 in the mRNA data of jejunal tissue. (**D**): Absolute loadings of the top features of Factor 1 in the mRNA data of liver tissue.

We applied MOFA (Multi-Omics Factor Analysis) to evaluate chicken samples, focusing on the key genes associated with jejunal RNA expression, liver RNA expression, and data from chicken assays. MOFA identified two factors that accounted for over 10% of the variance in the data. Factor 1 captured variability across all data modalities, while factor 2 was specific to the chicken data assay (Fig. 8A).

In the chicken data assay, factor 1 was linked to body weight at 6 weeks of age (BW6) (Fig. 8B). Furthermore, the analysis of the top weights in mRNA data showed that factor 1 was associated with *ZBTB46* in the jejunum and *WDR86* in the liver (Fig. 8C& D).

**Fig. 9:**
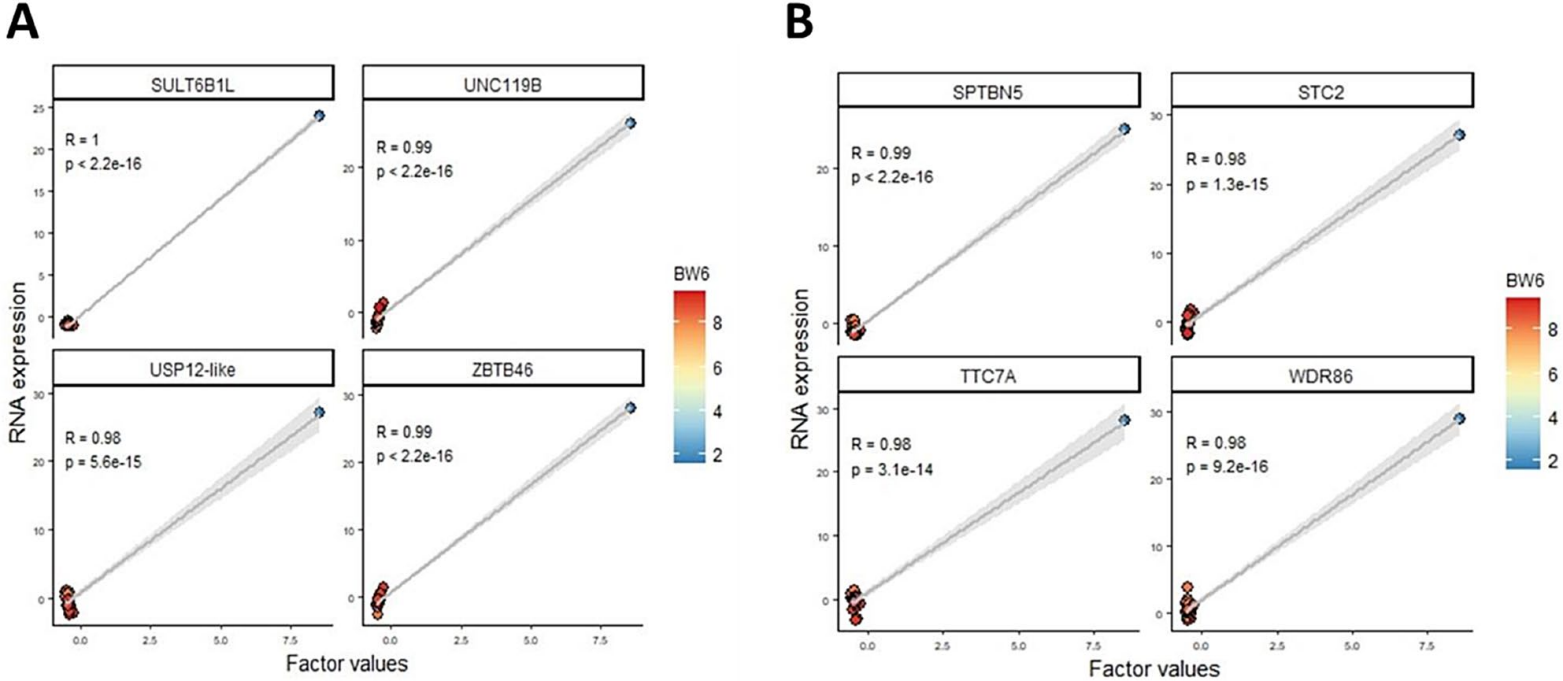
Molecular signature scatter plots. (**A**): Scatterplot displaying Factor 1 (x-axis) against expression values (y-axis) for the top four genes with the largest positive weight in the jejunal mRNA data. (**B**): Scatterplot of Factor 1 (x-axis) versus expression values (y-axis) for the top four genes with the largest positive weight in the liver mRNA data. Samples are color-coded based onBW6.

Figure 9 illustrates the molecular signatures within the mRNA data from the jejunum and liver, highlighting genes with significant positive weights. This analysis indicates that these genes exhibited higher expression levels in samples from younger birds, specifically at 6 weeks of age. In jejunal tissue, a significant correlation was observed between BW6 and the expression of genes, with the top four expressed genes being Sulfotransferase Family 6B Member 1 (*SULT6B1*), Ubiquitin Specific Peptidase 12-like (*USP12-like*), Unc-119 Lipid Binding Chaperone B (*UNC-119 B*), and Zinc Finger and BTB Domain Containing 46 (*ZBTB46*) (Fig. 9A). In liver tissue, a significant correlation was also found between weight at 6 weeks of age and gene expression, with the top four genes being Spectrin Beta Non-Erythrocytic 5 (*SPTBN5*), Stanniocalcin 2 (*STC2*), Tetratricopeptide Repeat Domain 7A (*TTC7A*), and WD Repeat-Containing Protein 86 (*WDR86*) (Fig. 9B). Additionally, we utilized the Reactome Pathway Knowledgebase (https://reactome.org) to identify genes involved in protein metabolism, specifically emphasizing *STC2* and *USP12*, which are recognized as key markers associated with Factor 1.

### Verification of DEGs using qRT-PCR

**Fig. 10:**
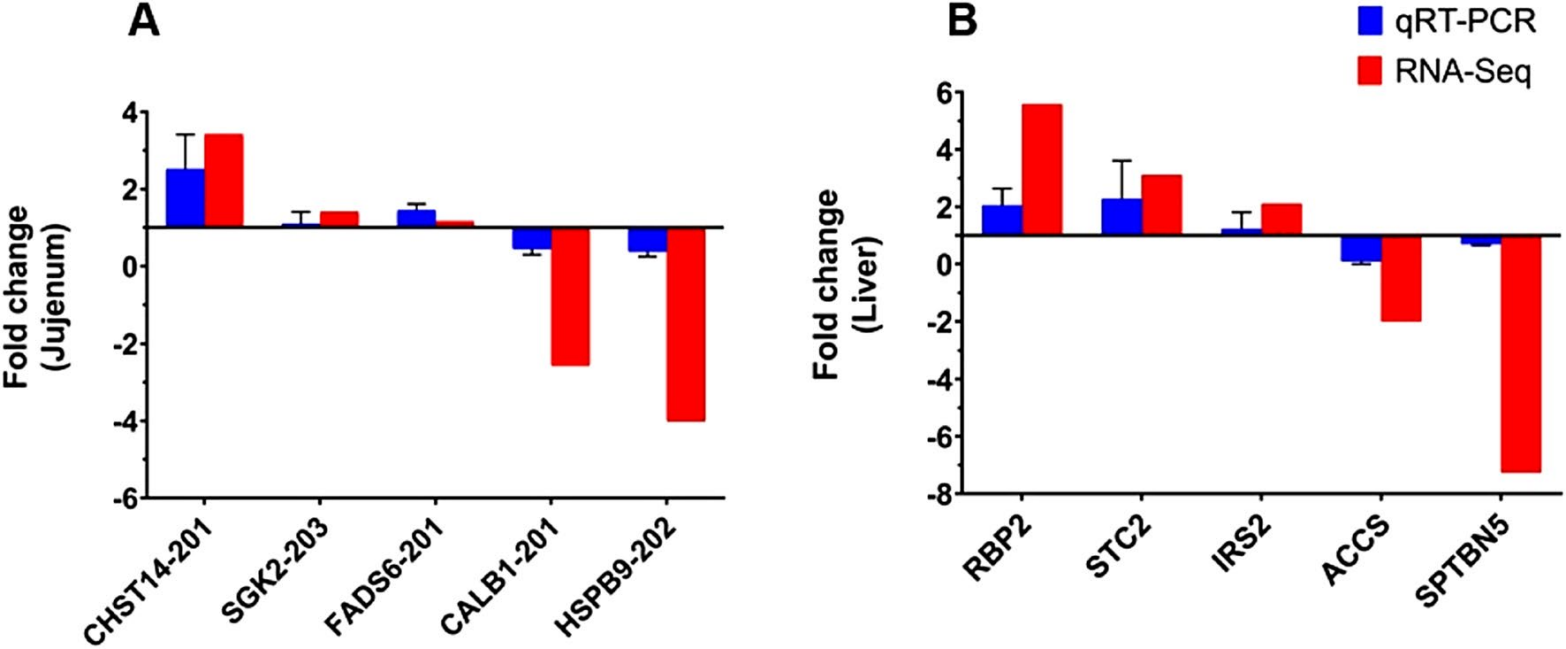
Quantitative real-time PCR (qRT-PCR) and RNA-Seq data of selected genes in (**A**) jejunum and (**B**) liver tissues from the top 10 males in both the high-weight and low-weight groups. Five randomly selected upregulated/ downregulated DEGs were selected for qPCR analysis and validation. Beta-actin served as the reference gene for qRT-PCR normalization. The mRNA expression levels for the selected genes were calculated using the 2 − △△CT method.

For validation, we randomly selected five DEGs for qRT-PCR analysis in jejunal and liver tissues from both the HW and LW groups. The primers used are presented in Table 1. The results indicated that the trends of relative mRNA expression levels of these selected genes were consistent with the findings from the transcriptome analysis (Fig. 10).

## Discussion

The body weight and daily weight gain values for Golden Montazah chickens are consistent with previous findings by Youssef et al.^43^, revealing significant differences in final body weight at 7 weeks despite similar feeding conditions. These variations can be attributed to the breed’s genetic diversity and lack of structured artificial selection among ten Egyptian chicken strains^43–46^. To explore the molecular changes between high-weight (HW) and low-weight (LW) groups, we employed RNA sequencing techniques to identify differentially expressed genes (DEGs) in both the jejunum and liver. Our research highlights the crucial roles of both the jejunum and liver in poultry health, and metabolism, emphasizing their complementary functions in regulating various biological processes relevant to growth performance.

In the jejunum, the upregulation of genes such as *CHST14* and *LOC429682* enhances mucosal defenses against toxins and pathogens, suggesting that high-weight broilers may exhibit stronger immune functionality^47–50^. Conversely, downregulated genes like* LOC772158* and HSPB9 in heavier birds may increase susceptibility to infections in lighter birds^51,52^. This relationship is supported by findings from Mishra and Jha^53^, indicating that enteric commensal bacteria produce reactive oxygen and nitrogen species, leading to intestinal inflammation and heightened heat shock protein (HSP) responses, which suggest that gene expression significantly affects the immune capacity of birds^54,55^.

GO analysis revealed six significant categories. Molecular functions involve binding and catalysis, while regulatory roles influencing macromolecular complexes essential for metabolic pathways and gene expression^56^. Cellular processes include cell communication, and cellular anatomical entities refer to cell parts as organelles and membranes^57^. Additionally, cellular components describe various cell structures, all of which highlight the complex roles of these genes in regulating essential biological processes in poultry. Enrichment in the cytokine-cytokine receptor interaction pathway further highlights the jejunum’s role in maintaining gut health, regulating immune responses, and promoting cell growth and differentiation, involving key genes such as *ACVR2B*, *GDF2*, and *TNFRSF13C*. *ACVR2B* mediates the effects of myostatin, which negatively regulates muscle mass, making this signaling pathway vital for muscle development and metabolic health. *GDF2* promotes cell growth and regeneration, supporting muscle tissue repair and angiogenesis, which are critical for nutrient supply and gut immune function. *TNFRSF13C* plays a key role in B-cell development and differentiation, enhancing B-cell survival and maturation for antibody production, while also influencing T-cell responses through immune interactions^58–60^. Together, these genes underscore the communication between the jejunum and systemic circulation through metabolic processes and immune interactions, emphasizing the connection between the jejunum and liver. Moreover, positive expressions in phosphatidylinositol and B cell receptor signaling pathways highlight the metabolic and immune regulation of jejunum^61,62^. Conversely, reduced expression in p53 and Homologous Recombination pathways in heavier birds suggest lower stress response activation, while lighter birds may be more prone to inflammation and barrier disruption^52,63–65^. Protein–protein interaction (PPI) analysis in the jejunum identified proteins (AHSG, FGG, ORM1, ITIH3, HRG) involved in immunity and gut homeostasis^66,67^. Lipid metabolism proteins (LIPC, ELOVL2) support energy production and storage, while AvBD9 and HAO2 contribute to antimicrobial defense and fatty acid metabolism, respectively. E1BX N4 regulates gut motility and appetite, impacting nutrient uptake efficiency. Given the jejunum’s high demand for energy and amino acids, these proteins are vital for efficient nutrient absorption and energy production in chickens^68,69^. These findings highlight the jejunum’s pivotal roles in digestion, nutrient absorption, immune regulation, and energy production. Notably, over 80% of jejunal mucosal cells are enterocytes, which support rapid protein turnover and growth^70^. Furthermore, Multi-Omics Factor Analysis (MOFA) established significant associations between weight at six weeks with the expression of *SULT6B1*, *USP12-like*, *UNC-119 B*, and *ZBTB46* in the jejunum. This highlights their roles in metabolism and immune responses, underscoring the jejunum’s integral role in overall poultry health^71–76^.

Additionally, our research highlights the complementary role of the liver in regulating growth and metabolic processes in poultry. The upregulation of *RBP2* and *STC2* genes enhances metabolic efficiency and immune function. *RBP2* facilitates vitamin A transport, binds unesterified fatty acids, and acts as a transcriptional regulator, while *STC2 *regulates glucose, phosphorus, and lipid metabolism^77–79^. These findings suggest that nutritional absorption and immune signaling from the jejunum directly impact the liver’s metabolic processes. Enhanced gene expression in heavier birds may contribute to improved metabolic, immune functions, as well as better growth performance. In contrast, the downregulation of *ACCS* and *SPTBN5* may indicate potential metabolic deficiencies that could hinder growth performance in lighter birds^75,80^.

Key GO categories in the liver highlight essential molecular functions, biological processes, and multicellular organismal activities that contribute to development and metabolism in chickens, with an emphasis on the importance of the cell periphery and plasma membrane in facilitating cellular communication^8,22,81–83^. The enrichment of the neuroactive ligand-receptor interaction pathway highlights its role in appetite regulation and energy metabolism by integrating hunger and satiety signals to modulate feeding behavior based on nutritional needs^84^. Key genes in this pathway, including *NPY*, *CHRM5*, *IAPP*, *GPR83*, *GPR25*, *MLNR*, regulate feed intake and energy balance^85,86^. For instance, *NPY* stimulates appetite, *CHRM5* impacts appetite and gastrointestinal motility, and *IAPP* regulates insulin secretion and appetite^87,88^. *GPR83L* is involved in behavioral regulation and immune modulation, whereas *GPR25* may contribute to metabolic processes. *MLNR* regulates gastrointestinal motility and gastric emptying, thus affecting feed intake efficiency^88,89^. The steroid biosynthesis pathway features *CYP24A1* and *LIPML5*, critical for steroid hormone regulation and lipid metabolism, respectively. Additionally, the Neurotrophin signaling pathway promotes cell survival and growth, during development^90,91^, while the Adherens Junctions pathway is essential for maintaining tissue integrity and cellular movement^92,93^. Negative expression patterns in the Ribosome and Protein Export pathways indicate potential challenges in protein synthesis among bird populations, potentially leading to impaired growth and development due to inadequate protein production^94^. These findings emphasize the liver’s critical role in managing energy balance and physiological functions in response to nutrients and immune signals from the jejunum. Moreover, lower weights in chickens may be linked to increased acute heat stress responses^95^, further underscoring the liver’s significance in lipid metabolism and overall health^23^. Thus, enhancing the interconnected genes and pathways is essential for improving liver function, which is vital for the growth and health of poultry. Liver PPI analysis has identified ten key hub proteins—NPY, RGS13, THEMIS, S100B, CALB1, AQP4, IAPP, PCSK1, LIPML5, CYP7A1— that are essential for appetite regulation, metabolism, and immune function. These proteins collectively highlight the interconnectedness of metabolic regulation and immune responses within the liver. Specifically, NPY stimulates appetite and energy homeostasis, RGS13 modulates G protein-coupled receptor signaling, and THEMIS influences T-cell development. Other proteins, such as S100B, CALB1, AQP4, IAPP, PCSK1, LIPML5, and CYP7A1, play critical roles in inflammation, calcium homeostasis, osmotic balance, glucose metabolism, prohormone processing, lipid metabolism, and bile acid synthesis^86,96^. Overall, these findings underscore the liver’s vital role in maintaining energy balance and metabolic activity^97^.

MOFA has linked the genes *SPTBN5*, *STC2*, *TTC7A*, and *WDR86* in the liver to weight at six weeks of age, indicating their roles in metabolism and immune responses^75,98,99^. These genes may influence critical physiological processes, particularly in protein and lipid metabolism essential for growth and development. Additionally, a predicted association was found between *ZBTB46* in the jejunum and *WDR86* in the liver. *ZBTB46*, a transcription factor in group 3 innate lymphoid cells (ILC3s), regulates inflammatory responses and promotes the secretion of antimicrobial peptides via IL-22 production, protecting against bacterial invasion and aiding nutrient absorption^90,91^. While specific information about *WDR86* is limited, it is known to play a vital role in various cellular processes, including metabolism and immune responses, crucial for liver function and energy homeostasis. The predicted association between *ZBTB46* and *WDR86* illustrates the complex relationship between gut health and liver metabolism and immune function, offering insights into how nutritional and immune factors work together to support overall health in chickens. The identification of *STC2* and *USP12* as markers related to Factor 1 highlights their significant roles in poultry production. *STC2* is known for its diverse functions, including calcium regulation, stress-induced cell survival, and potential growth modulation^79^. *USP12*, a deubiquitinating enzyme, regulates protein stability, function, and degradation, impacting immune signaling and stress responses^100^. Dysregulation of *STC2* can disrupt lipid metabolism, affecting nutrient absorption and immune function, potentially intensified by alterations in protein stability controlled by *USP12*. Together, *STC2* and *USP12* influence essential factors like growth rates, feed efficiency, and skeletal development, which are critical for optimal chicken production. Their involvement in stress responses and immune regulation suggests they may improve disease resistance and facilitate environmental adaptation by modulating cellular and metabolic pathways. Overall, these characteristics position *STC2* and *USP12* as valuable genetic markers for selective breeding programs aimed at improving poultry health and productivity.

Although transcriptomic profiling provides a comprehensive overview of gene expression changes, it remains largely descriptive and cannot establish causal relationships on its own. Limitations such as tissue heterogeneity and potential confounders can affect interpretation. To address these issues, future studies integrating multi-omics approaches, particularly proteomics, are proposed to validate whether transcriptional changes correspond to alterations at the protein level, thereby enabling more robust mechanistic insights and broader biological implications to better understand complex traits like body weight regulation.

## Conclusion

Our findings emphasize the intricate interplay between the jejunum and liver in regulating growth, metabolism, and immune function in poultry. The gene expression patterns observed in both organs are vital for maintaining optimal health and performance, with key genes such as *ZBTB46*, *STC2*, *WDR86*, and *USP12* playing central roles in essential biological processes. The upregulation of immune-related genes in the jejunum appears to bolster mucosal defenses and enhance nutrient absorption, while gene activity in the liver contributes significantly to metabolic efficiency and appetite regulation. These interconnected pathways highlight the importance of a coordinated response between the gut and liver, which collectively support growth and bolster resilience against environmental stressors. The insights gained from this research not only deepen our understanding of the molecular mechanisms underpinning poultry health but also open new avenues for targeted nutritional and genetic interventions. Such strategies hold promises for improving overall productivity, promoting health, and advancing sustainability in the poultry industry—ultimately leading to more resilient and profitable production systems.

## Supplementary Information

Below is the link to the electronic supplementary material.

Supplementary Material 1

Supplementary Material 2

Supplementary Material 3

Supplementary Material 4

Supplementary Material 5

## Data Availability

All raw data from RNA sequencing were uploaded to the National Center for Biotechnology Information (NCBI) and can be found under BioProject ID PRJNA1230854, accessed on March 3, 2025.

## References

[1] P Barrow, V Nair, S Baigent, R Atterbury. & M Clark. Poultry health: a guide for professionals. (Cabi, 2021)

[2] Gheyas, A. A. et al. Integrated environmental and genomic analysis reveals the drivers of local adaptation in African indigenous chickens. *Mol. Biol. Evol.* **38**, 4268–4285 (2021). 34021753 10.1093/molbev/msab156 PMC8476150

[3] Mpenda, F. N., Schilling, M. A., Campbell, Z., Mngumi, E. B. & Buza, J. The genetic diversity of local african chickens: A potential for selection of chickens resistant to viral infections. *J. Appl. Poultry Res.* **28**(1), 1–12 (2019).

[4] El-Komy, E. M. et al. Genetic diversity, population structure and their association with body weight in Egyptian chicken strains. *J. World’s Poult. Res.* **13**, 440–449 (2023).

[5] Zhang, X. et al. Caecal microbiota could effectively increase chicken growth performance by regulating fat metabolism. *Microb. Biotechnol.* **15**, 844–861 (2022). 34264533 10.1111/1751-7915.13841 PMC8913871

[6] Chen, F. et al. Transcriptome analysis of differentially expressed genes related to the growth and development of the Jinghai yellow chicken. *Genes (Basel).* **10**, 539 (2019). 31319533 10.3390/genes10070539 PMC6678745

[7] Liu, L. et al. Transcriptional insights into key genes and pathways controlling muscle lipid metabolism in broiler chickens. *BMC Genomics* **20**(1), 863 (2019). 31729950 10.1186/s12864-019-6221-0 PMC6858653

[8] Kumar, H. et al. RNA seq analyses of chicken reveals biological pathways involved in acclimation into different geographical locations. *Sci. Rep.* **10**, 19288 (2020). 33159110 10.1038/s41598-020-76234-8 PMC7648748

[9] Xing, S. et al. RNA-Seq analysis reveals hub genes involved in chicken intramuscular fat and abdominal fat deposition during development. *Front. Genet.* **11**, 1009 (2020). 33117416 10.3389/fgene.2020.01009 PMC7493673

[10] Yang, L. et al. Identification of key genes and pathways associated with feed efficiency of native chickens based on transcriptome data via bioinformatics analysis. *BMC Genomics* **21**, 292 (2020). 32272881 10.1186/s12864-020-6713-y PMC7146967

[11] Zhang, G. et al. Study on the transcriptome for breast muscle of chickens and the function of key gene RAC2 on fibroblasts proliferation. *BMC Genomics* **22**, 157 (2021). 33676413 10.1186/s12864-021-07453-0 PMC7937270

[12] Perlas, A. et al. Dual Host and Pathogen RNA-Seq Analysis Unravels Chicken Genes Potentially Involved in Resistance to Highly Pathogenic Avian Influenza Virus Infection. *Front. Immunol.* **12**, 800188 (2021). 35003125 10.3389/fimmu.2021.800188 PMC8727699

[13] Dar, M. A. et al. Identification of SNPs related to salmonella resistance in chickens using RNA-Seq and integrated bioinformatics approach. *Genes (Basel).* **14**, 1283 (2023). 37372463 10.3390/genes14061283 PMC10297900

[14] Tan, X. et al. Large-scale genomic and transcriptomic analyses elucidate the genetic basis of high meat yield in chickens. *J. Adv. Res.* **55**, 1–16 (2024). 36871617 10.1016/j.jare.2023.02.016 PMC10770282

[15] Ravindran, V. & Abdollahi, M. R. Nutrition and digestive physiology of the broiler chick: State of the art and outlook. *Animals (Basel).* **11**, 2795 (2021). 34679817 10.3390/ani11102795 PMC8532940

[16] Song, J. et al. The effect of Epigallocatechin-3-gallate on small intestinal morphology, antioxidant capacity and anti-inflammatory effect in heat-stressed broilers. *J. Anim. Physiol. Anim. Nutr. (Berl)* **103**, 1030–1038 (2019). 30702179 10.1111/jpn.13062

[17] Sinpru, P. et al. Jejunal transcriptomic profiling for differences in feed conversion ratio in slow-growing chickens. *Animals (Basel).* **11**, 2606 (2021). 34573572 10.3390/ani11092606 PMC8470203

[18] Kalra, A., Yetiskul, E., Wehrle, C. & Tuma, F. (StatPearls Publishing, 2023). 30571059

[19] Aimee, Y. Y. et al. Impaired physiological responses to chronic hypoxia in mice partially deficient for hypoxia-inducible factor 1α. *J. Clin. Investig.* **103**, 691–696 (1999). 10074486 10.1172/JCI5912 PMC408131

[20] Trefts, E., Williams, A. S. & Wasserman, D. H. Exercise and the regulation of hepatic metabolism. *Prog. Mol. Biol. Transl. Sci.* **135**, 203–225 (2015). 26477916 10.1016/bs.pmbts.2015.07.010 PMC4826571

[21] Bertocchi, M. et al. Exploring differential transcriptome between jejunal and cecal tissue of broiler chickens. *Animals (Basel).* **9**, 221 (2019). 31067716 10.3390/ani9050221 PMC6562892

[22] Kim, H. et al. Transcriptomic response under heat stress in chickens revealed the regulation of genes and alteration of metabolism to maintain homeostasis. *Animals (Basel).* **11**, 2241 (2021). 34438700 10.3390/ani11082241 PMC8388523

[23] Li, H. et al. Transcriptome profile of liver at different physiological stages reveals potential mode for lipid metabolism in laying hens. *BMC Genomics* **16**, 1–13 (2015). 26452545 10.1186/s12864-015-1943-0 PMC4600267

[24] El-Sheikh, T.M.J.E.P.S.J. Effect of continuous and intermittent high ambient temperature on growing males of gimmizah and golden-montazah chicken performanCE. 36, 725–741 (2016).

[25] Hosny, F. A. The structure and importance of the commercial and village based poultry systems in Egypt. *Poult. Sect. Count. Rev* **1**, 39 (2006).

[26] Bélanger, J. & Pilling, D. The state of the world’s biodiversity for food and agriculture. (FAO;, 2019).

[27] Wu, P. et al. Transcriptome profile analysis of leg muscle tissues between slow-and fast-growing chickens. *PLoS ONE* **13**(11), e0206131 (2018). 30403718 10.1371/journal.pone.0206131 PMC6221307

[28] Ewels, P., Magnusson, M., Lundin, S. & Kaller, M. MultiQC: Summarize analysis results for multiple tools and samples in a single report. *Bioinformatics* **32**, 3047–3048 (2016). 27312411 10.1093/bioinformatics/btw354 PMC5039924

[29] Kim, D., Langmead, B. & Salzberg, S. L. HISAT: A fast spliced aligner with low memory requirements. *Nat. Methods.* **12**, 357–360 (2015). 25751142 10.1038/nmeth.3317 PMC4655817

[30] Liao, Y., Smyth, G. K. & Shi, W. featureCounts: An efficient general purpose program for assigning sequence reads to genomic features. *Bioinformatics* **30**, 923–930 (2014). 24227677 10.1093/bioinformatics/btt656

[31] Robinson, M. D., McCarthy, D. J. & Smyth, G. K. edgeR: a Bioconductor package for differential expression analysis of digital gene expression data. *Bioinformatics* **26**, 139–140 (2010). 19910308 10.1093/bioinformatics/btp616 PMC2796818

[32] Wickham, H., Chang, W. & Wickham, M.H.J.C.E.D.V.U.T.G.O.G.V. Package ‘ggplot2’. **2**, 1–189 (2016).

[33] Kolde, R. (R-Project, 2019).

[34] Raudvere, U. et al. g:Profiler: a web server for functional enrichment analysis and conversions of gene lists (2019 update). *Nucleic Acids Res.* **47**, W191–W198 (2019). 31066453 10.1093/nar/gkz369 PMC6602461

[35] Ge, S. X., Jung, D. & Yao, R. ShinyGO: A graphical gene-set enrichment tool for animals and plants. *Bioinformatics* **36**, 2628–2629 (2020). 31882993 10.1093/bioinformatics/btz931 PMC7178415

[36] Bertocchi, M. et al. In ovo Injection of a Galacto-Oligosaccharide Prebiotic in Broiler Chickens Submitted to Heat-Stress: Impact on Transcriptomic Profile and Plasma Immune Parameters. *Animals (Basel).* **9**, 1067 (2019). 10.3390/ani9121067 PMC6940861 31810282

[37] Hoshikawa, M. et al. NK cell and IFN signatures are positive prognostic biomarkers for resectable pancreatic cancer. *Biochem Biophys Res Commun.* **495**, 2058–2065 (2018). 29253566 10.1016/j.bbrc.2017.12.083

[38] Mering, C. V. et al. STRING: a database of predicted functional associations between proteins. *Nucleic acids res.* **31**, 258–261 (2003). 12519996 10.1093/nar/gkg034 PMC165481

[39] Kohl, M., Wiese, S. & Warscheid, B. Cytoscape: Software for visualization and analysis of biological networks. *Methods Mol. Biol.* **696**, 291–303 (2011). 21063955 10.1007/978-1-60761-987-1_18

[40] Argelaguet, R. et al. Multi-omics factor analysis—a framework for unsupervised integration of multi-omics data sets. *Mol. sys. biol.* **14**, e8124 (2018). 10.15252/msb.20178124 PMC6010767 29925568

[41] Fabregat, A. et al. The reactome pathway knowledgebase. *Nucleic Acids Res.* **46**, D649–D655 (2018). 29145629 10.1093/nar/gkx1132 PMC5753187

[42] Livak, K. J. & Schmittgen, T. D. Analysis of relative gene expression data using real-time quantitative PCR and the 2− ΔΔCT method. *Methods* **25**(4), 402–408 (2001). 11846609 10.1006/meth.2001.1262

[43] Youssef, S., Yassein, D., El-Bahy, N. M. & Faddle, A. J. P. S. A comparative studies among golden montazah, el-salam and fayoumi chickens. 1-response to acute heat stress as early heat conditioning procedure. *Egypt. Egypt. Poult. Sci.* **34**, 1075–1097 (2014).

[44] Eltanany, M., Philipp, U., Weigend, S. & Distl, O. Genetic diversity of ten Egyptian chicken strains using 29 microsatellite markers. *Anim. Genet.* **42**, 666–669 (2011). 22035011 10.1111/j.1365-2052.2011.02185.x

[45] Ashour, A., Badwi, Y., El-Karim, A., Ragaa, E. J. J. O. A. & Production, P. Effect of selection for body weight on egg production, egg quality, fertility and hatchability traits in El-salam chicken strain in Egypt. *J. Anim. Poult. Prod.* **6**, 781–796 (2015).

[46] Elbeltagy, A. R. et al. Natural selection footprints among African chicken breeds and village ecotypes. *Front. Genet.* **10**, 376 (2019). 31139205 10.3389/fgene.2019.00376 PMC6518202

[47] Hubbard, S. J. et al. Transcriptome analysis for the chicken based on 19,626 finished cDNA sequences and 485,337 expressed sequence tags. *Genome Res.* **15**, 174–183 (2005). 15590942 10.1101/gr.3011405 PMC540287

[48] Min, W. et al. Expressed sequence tag analysis of Eimeria-stimulated intestinal intraepithelial lymphocytes in chickens. *Mol Biotechnol.* **30**, 143–150 (2005). 15920284 10.1385/MB:30:2:143

[49] McKee, T. J., Perlman, G., Morris, M. & Komarova, S. V. Extracellular matrix composition of connective tissues: A systematic review and meta-analysis. *Sci. Rep.* **9**, 10542 (2019). 31332239 10.1038/s41598-019-46896-0 PMC6646303

[50] Begolli, G., Markovic, I., Knezevic, J. & Debeljak, Z. Carbohydrate sulfotransferases: a review of emerging diagnostic and prognostic applications. *Biochem. Med (Zagreb)* **33**, 030503 (2023). 37545696 10.11613/BM.2023.030503 PMC10373059

[51] Al-Zghoul, M. B. & El-Bahr, S. M. Basal and dynamics mRNA expression of muscular HSP108, HSP90, HSF-1 and HSF-2 in thermally manipulated broilers during embryogenesis. *BMC Vet. Res.* **15**, 83 (2019). 30849975 10.1186/s12917-019-1827-7 PMC6408791

[52] Abdel-Kafy, E. M. et al. Gut microbiota, intestinal morphometric characteristics, and gene expression in relation to the growth performance of chickens. *Animals (Basel)* **12**, 3474 (2022). 36552394 10.3390/ani12243474 PMC9774407

[53] Mishra, B. & Jha, R. Oxidative stress in the poultry gut: Potential challenges and interventions. *Front. Vet. Sci.* **6**, 60 (2019). 30886854 10.3389/fvets.2019.00060 PMC6409315

[54] Alqazlan, N. et al. Transcriptomics of chicken cecal tonsils and intestine after infection with low pathogenic avian influenza virus H9N2. *Sci. Rep.* **11**, 20462 (2021). 34650121 10.1038/s41598-021-99182-3 PMC8517014

[55] Hu, C. et al. Heat shock proteins: Biological functions, pathological roles, and therapeutic opportunities. *MedComm (2020)* **3**, e161 (2022). 35928554 10.1002/mco2.161 PMC9345296

[56] Pan, Z. et al. An atlas of regulatory elements in chicken: A resource for chicken genetics and genomics. *Sci. Adv.* **9**, eade1204 (2023). 37134160 10.1126/sciadv.ade1204 PMC10156120

[57] Vanamamalai, V. K., Priyanka, E., Kannaki, T. R. & Sharma, S. Integrative study of chicken lung transcriptome to understand the host immune response during Newcastle disease virus challenge. *Front. Cell. Infect. Microbiol.* **14**, 1368887 (2024). 39290979 10.3389/fcimb.2024.1368887 PMC11405381

[58] Lee, S.-J. et al. Regulation of muscle growth by multiple ligands signaling through activin type II receptors. *Proc. Natl. Acad. Sci. U. S.* **102**, 18117–18122 (2005). 10.1073/pnas.0505996102 PMC1306793 16330774

[59] Mackay, F. & Schneider, P. TACI, an enigmatic BAFF/APRIL receptor, with new unappreciated biochemical and biological properties. *Cytokine Growth Factor Rev.* **19**, 263–276 (2008). 18514565 10.1016/j.cytogfr.2008.04.006

[60] Black, A. N. et al. A Highly Contiguous and Annotated Genome Assembly of the Lesser Prairie-Chicken (Tympanuchus pallidicinctus). *Genome Biol. Evol.* **15**, evad043 (2023). 36916502 10.1093/gbe/evad043 PMC10118296

[61] Erf, G. F. Cell-mediated immunity in poultry. *Poult. Sci.* **83**, 580–590 (2004). 15109055 10.1093/ps/83.4.580

[62] Mazet, F., Tindall, M. J., Gibbins, J. M. & Fry, M. J. A model of the PI cycle reveals the regulating roles of lipid-binding proteins and pitfalls of using mosaic biological data. *Sci. Rep.* **10**, 13244 (2020). 32764630 10.1038/s41598-020-70215-7 PMC7414024

[63] Michel, B., Boubakri, H., Baharoglu, Z., LeMasson, M. & Lestini, R. Recombination proteins and rescue of arrested replication forks. *DNA Repair (Amst).* **6**, 967–980 (2007). 17395553 10.1016/j.dnarep.2007.02.016

[64] Levine, A. J. The many faces of p53: something for everyone. *J. Mol. Cell Biol.* **11**, 524–530 (2019). 30925588 10.1093/jmcb/mjz026 PMC6736316

[65] Zhang, X. et al. Caecal microbiota could effectively increase chicken growth performance by regulating fat metabolism. *Microbial Biotechnol.* **15**(3), 844–861 (2022). 10.1111/1751-7915.13841 PMC8913871 34264533

[66] Tian, W.-D. et al. Proteomic identification of alpha-2-HS-glycoprotein as a plasma biomarker of hypopharyngeal squamous cell carcinoma. *Int. J. Clin. Exp. Pathol.* **8**, 9021 (2015). 26464644 PMC4583876

[67] Manni, M., Berkeley, M. R., Seppey, M., Simao, F. A. & Zdobnov, E. M. BUSCO update: Novel and streamlined workflows along with broader and deeper phylogenetic coverage for scoring of eukaryotic, prokaryotic, and viral genomes. *Mol. Biol. Evol.* **38**, 4647–4654 (2021). 34320186 10.1093/molbev/msab199 PMC8476166

[68] Kim, D. Y., Lim, B., Kim, J. M. & Kil, D. Y. Integrated transcriptome analysis for the hepatic and jejunal mucosa tissues of broiler chickens raised under heat stress conditions. *J. Anim. Sci. Biotechnol.* **13**, 79 (2022). 35843965 10.1186/s40104-022-00734-y PMC9290309

[69] Zhao, W. et al. Ligand recognition and activation of neuromedin U receptor 2. *Nat. Commun.* **13**, 7955 (2022). 36575163 10.1038/s41467-022-34814-4 PMC9794833

[70] Zhu, Q. et al. RNA sequencing transcriptomics and metabolomics in three poultry breeds. *Sci. Data.* **10**, 594 (2023). 37679362 10.1038/s41597-023-02505-4 PMC10484955

[71] Maduro, M. F., Gordon, M., Jacobs, R. & Pilgrim, D. B. J. J. O. N. The UNC-119 family of neural proteins is functionally conserved between humans. *Drosophila and C. elegans. J. Neurogenet.* **13**, 191–212 (2000). 10858820 10.3109/01677060009084494

[72] Constantine, R., Zhang, H., Gerstner, C. D., Frederick, J. M. & Baehr, W. Uncoordinated (UNC)119: Coordinating the trafficking of myristoylated proteins. *Vision Res.* **75**, 26–32 (2012). 23000199 10.1016/j.visres.2012.08.012 PMC3514684

[73] Chen, S., Liu, Y. & Zhou, H. Advances in the development ubiquitin-specific peptidase (USP) inhibitors. *Int. J. Mol. Sci.* **22**, 4546 (2021). 33925279 10.3390/ijms22094546 PMC8123678

[74] Yi, M., Negishi, M. & Lee, S. J. Estrogen sulfotransferase (SULT1E1): Its molecular regulation, polymorphisms, and clinical perspectives. *J. Pers. Med.* **11**, 194 (2021). 33799763 10.3390/jpm11030194 PMC8001535

[75] Farrell, C. M. et al. RefSeq functional elements as experimentally assayed nongenic reference standards and functional interactions in human and mouse. *Genome res.* **32**, 175–188 (2022). 34876495 10.1101/gr.275819.121 PMC8744684

[76] W Zhou et al. ZBTB46 defines and regulates ILC3s that protect the intestine. **609**, 159–165 (2022). 10.1038/s41586-022-04934-4 PMC9528687 35831503

[77] Hebiguchi, T. et al. Massive bowel resection upregulates the intestinal mRNA expression levels of cellular retinol-binding protein II and apolipoprotein A-IV and alters the intestinal vitamin A status in rats. *Int. J. Mol. Med.* **35**, 724–730 (2015). 25585692 10.3892/ijmm.2015.2066

[78] Li, S. et al. The significance of Stanniocalcin 2 in malignancies and mechanisms. *Bioengineered* **12**, 7276–7285 (2021). 34612765 10.1080/21655979.2021.1977551 PMC8806499

[79] Cao, Y. et al. stc2 inhibits hepatic lipid synthesis and correlates with intramuscular fatty acid composition, body weight and carcass traits in chickens. *Animals (Basel).* **14**, 383 (2024). 38338026 10.3390/ani14030383 PMC10854843

[80] Khan, S., Alvi, A. F., Saify, S., Iqbal, N. & Khan, N. A. The ethylene biosynthetic enzymes, 1-aminocyclopropane-1-carboxylate (ACC) synthase (ACS) and ACC Oxidase (ACO): The less explored players in abiotic stress tolerance. *Biomolecules* **14**, 90 (2024). 38254690 10.3390/biom14010090 PMC10813531

[81] Mossio, M., Montevil, M. & Longo, G. Theoretical principles for biology: Organization. *Prog. Biophys Mol. Biol.* **122**, 24–35 (2016). 27521451 10.1016/j.pbiomolbio.2016.07.005

[82] Alessandroni, L., Sagratini, G. & Gagaoua, M. Proteomics and bioinformatics analyses based on two-dimensional electrophoresis and LC-MS/MS for the primary characterization of protein changes in chicken breast meat from divergent farming systems: Organic versus antibiotic-free. *Food Chem (Oxf).* **8**, 100194 (2024). 38298469 10.1016/j.fochms.2024.100194 PMC10828576

[83] Xie, X. et al. Danzhou chicken: a unique genetic resource revealed by genome-wide resequencing data. *Poult. Sci.* **103**, 103960 (2024). 38964270 10.1016/j.psj.2024.103960 PMC11278292

[84] Morton, G. J., Meek, T. H. & Schwartz, M. W. Neurobiology of food intake in health and disease. *Nat. Rev. Neurosci.* **15**, 367–378 (2014). 24840801 10.1038/nrn3745 PMC4076116

[85] Akter, R. et al. Islet Amyloid Polypeptide: Structure, Function, and Pathophysiology. *J. Diabetes Res.* **2016**, 2798269 (2016). 26649319 10.1155/2016/2798269 PMC4662979

[86] Greene, E. S., Abdelli, N., Dridi, J. S. & Dridi, S. Avian Neuropeptide Y: Beyond Feed Intake Regulation. *Vet. Sci.* **9**, 171 (2022). 35448669 10.3390/vetsci9040171 PMC9028514

[87] Pedersen, J.E., Bergqvist, C.A. & Larhammar, D. Evolution of the Muscarinic Acetylcholine Receptors in Vertebrates. *eNeuro.* **5** (2018) 10.1523/ENEURO.0340-18.2018 PMC6298421 30564629

[88] Saroz, Y., Kho, D. T., Glass, M., Graham, E. S. & Grimsey, N. L. Cannabinoid Receptor 2 (CB(2)) Signals via G-alpha-s and Induces IL-6 and IL-10 Cytokine Secretion in Human Primary Leukocytes. *ACS Pharmacol. Transl. Sci.* **2**, 414–428 (2019). 32259074 10.1021/acsptsci.9b00049 PMC7088898

[89] Kitazawa, T., Teraoka, H. & Kaiya, H. J. E. J. The chicken is an interesting animal for study of the functional role of ghrelin in the gastrointestinal tract. *Endocr. J.* **64**, S5–S9 (2017). 28652545 10.1507/endocrj.64.S5

[90] Arevalo, J. C. & Wu, S. H. Neurotrophin signaling: many exciting surprises!. *Cell Mol. Life Sci.* **63**, 1523–1537 (2006). 16699811 10.1007/s00018-006-6010-1 PMC11135985

[91] Skaper, S. D. The neurotrophin family of neurotrophic factors: an overview. *Methods Mol. Biol.* **846**, 1–12 (2012). 22367796 10.1007/978-1-61779-536-7_1

[92] Irie, K., Shimizu, K., Sakisaka, T., Ikeda, W. & Takai, Y. in Seminars in cell & developmental biology, Vol. 15 643–656 (Elsevier, 2004). 10.1016/j.semcdb.2004.09.002 15561584

[93] Harris, T. J. & Tepass, U. Adherens junctions: from molecules to morphogenesis. *Nat. Rev. Mol. Cell. Biol.* **11**, 502–514 (2010). 20571587 10.1038/nrm2927

[94] Warner, J. R. & McIntosh, K. B. J. M. C. How common are extraribosomal functions of ribosomal proteins?. *Mol. cell.* **34**, 3–11 (2009). 19362532 10.1016/j.molcel.2009.03.006 PMC2679180

[95] Chauhan, S. S., Rashamol, V. P., Bagath, M., Sejian, V. & Dunshea, F. R. Impacts of heat stress on immune responses and oxidative stress in farm animals and nutritional strategies for amelioration. *Int. J. Biometeorol.* **65**, 1231–1244 (2021). 33496873 10.1007/s00484-021-02083-3

[96] Yang, C. et al. Function and regulation of RGS family members in solid tumours: a comprehensive review. *Cell Commun Signal.* **21**, 316 (2023). 37924113 10.1186/s12964-023-01334-7 PMC10623796

[97] Luo, X., Guo, J., Zhang, J., Ma, Z. & Li, H. Overview of chicken embryo genes related to sex differentiation. *PeerJ* **12**, e17072 (2024). 38525278 10.7717/peerj.17072 PMC10959104

[98] Jardine, S., Dhingani, N. & Muise, A. M. TTC7A: Steward of Intestinal Health. *Cell Mol Gastroenterol Hepatol.* **7**, 555–570 (2019). 30553809 10.1016/j.jcmgh.2018.12.001 PMC6406079

[99] McGarvey, K. M. et al. Mouse genome annotation by the RefSeq project. *Mamm. Genome.* **26**, 379–390 (2015). 26215545 10.1007/s00335-015-9585-8 PMC4602073

[100] Niu, K. et al. Spotlights on ubiquitin-specific protease 12 (USP12) in diseases: from multifaceted roles to pathophysiological mechanisms. *J. Transl. Med.* **21**, 665 (2023). 37752518 10.1186/s12967-023-04540-6 PMC10521459

